# Dating Violence and Mental Health in Emerging Adulthood

**DOI:** 10.3390/healthcare11243172

**Published:** 2023-12-15

**Authors:** M. Pilar Matud, D. Estefanía Hernández-Lorenzo, Demelsa Fortes, Ignacio Ibáñez

**Affiliations:** Department of Clinical Psychology, Psychobiology and Methodology, Universidad de La Laguna, 38200 San Cristobal de La Laguna, Spain; dhernanl@ull.edu.es (D.E.H.-L.); demel81@hotmail.com (D.F.); iibanez@ull.edu.es (I.I.)

**Keywords:** dating violence, emerging adulthood, gender, mental symptoms, life satisfaction, self-esteem, traditional role attitudes, masculine/instrumental trait, feminine/expressive trait

## Abstract

Dating violence constitutes a serious social and health problem. This study aims to increase knowledge on dating violence in emerging adulthood by analysing the relevance of gender and of having or not having a current partner in the victimization and perpetration of such violence. It also analyses the association between dating violence and mental health, as well as the relevance of traditional gender role attitudes and the internalization of feminine/expressive and masculine/instrumental traits in the victimization and perpetration of such types of violence. The participants were 930 Spanish emerging adults who were assessed by six self-report questionnaires and scales. Men reported more psychological and physical violence victimization and physical violence perpetration than women, and women and men without a current partner reported more psychological and sexual violence than women and men with a current partner. Dating violence victimization was associated with more mental symptomatology, less life satisfaction, and lower self-esteem in men with a current partner and in women without a current partner. The main predictor of dating violence victimization was dating violence perpetration, and the main predictor of dating violence perpetration was victimization by such violence. More traditional gender role attitudes also predicted greater victimization and perpetration of dating violence, except among women without a current partner.

## 1. Introduction

Dating violence among adolescents and young adults is considered to be any type of intentional aggression by one partner against the other during dating [1]. It is a type of intimate partner violence (IPV). IPV refers to aggression or abuse that occurs in a romantic relationship and refers to both current and former spouses and dating partners [2]. Whereas no consensus exists on the definition of dating violence [3], such a term refers to violence within an unmarried partnership in the context of dating or courtship, generally referring to adolescents or young adults [3,4,5,6].

Although IPV can vary in terms of frequency and severity [2], IPV can comprise physical violence, sexual violence, stalking, and psychological aggression, including coercive tactics, by a current or a former intimate partner [7]. Although the various forms of aggression and violence are often associated, they are generally grouped into three broad categories: physical, psychological, and sexual [1,8]. Physical violence is defined as the intentional use of physical force with the potential to cause death, disability, injury, or harm. It covers a variety of behaviors, including scratching, pushing, shoving, punching, kicking, choking, slapping, hitting, burning, throwing objects, and using a weapon (gun, knife, or any other object) [7,8,9]. Psychological violence refers to the intentional use of nonverbal and verbal communication to mentally or emotionally harm and/or exert control over a partner. It can include a wide range of behaviors and coercive tactics, ranging from insulting, humiliation, criticizing, and name-calling to isolating the partner from friends or family, controlling partner behaviors, and threatening behavior [7,8,9,10]. Sexual violence is defined as “a sexual act that is committed or attempted by another person without freely given consent of the victim or against someone who is unable to consent or refuse” [7] (p. 11). IPV occurs across the lifespan, is a serious public health problem [2,11], and has many social and individual costs [2]. Dating violence is a very common phenomenon that, in addition to having serious health consequences, has been associated with intimate partner violence in adulthood [12].

Dating violence is a significant problem given its high prevalence and impacts on victims’ health and well-being, although research has shown that prevalence rates are widely variable [1,8,13]. In a systematic review study on the prevalence of dating violence in adolescents and young people, Rubio-Garay et al. [1] found that the percentages ranged from 8.5% to 95.5% in psychological violence victimization, from 0.4% to 57.3% in physical violence victimization, and from 0.1% to 64.4% in sexual violence victimization. The perpetration of psychological violence varied from 4.2% to 97%, whereas as regards the perpetration of physical violence, variation was from 3.8% to 41.9%, and from 1.2% to 58.8% in case of the perpetration of sexual violence. Moreover, in many of these studies, violence was bidirectional. The results of this review suggest a higher prevalence of perpetration of psychological violence by women and of sexual violence by men, as well as greater victimization by psychological and sexual violence in women [1]. Although “there exists a large body of research analysing gender differences with regard to dating aggression, findings have historically been mixed and inconclusive” [8] (p. 187). Meta-analysis and systematic review of the violence rates in dating relationships in adolescents and young adults have shown great heterogeneity among studies; although both males and females are generally found to be involved in violence perpetration and victimization, rates vary according to the type of violence and its severity [8,14], and according to life course stage [15]. There is evidence that in contexts of most serious violence, sexual violence included, women were identified as the main victims [14]. Research has consistently shown that men are more likely to inflict injury [8,16], whereas it is more common for women to suffer from injuries [8].

There are several possible explanations for prevalence rate fluctuations in dating violence, including definitional and measurement issues [3,13,15,16,17], as prevalence rates vary depending on the definitional criteria adopted for specific research [3,17]. The existence of selection bias and the reliability of reporting have also been cited [3,8,17]. One issue that has been considered is the degree to which women and men differentially participate in research on interpersonal violence, whose evidence reveals that women participate more often than men. Since male perpetration toward females is generally viewed as less acceptable than female perpetration, male perpetrators may intentionally not participate in dating violence research; in addition, men who do participate in such research may not accurately report or may minimize their violent behavior [3,8]. There is also evidence that women report rates of their own perpetration more often than men; furthermore, women appear to be more willing to report their perpetration of violent acts than to report their own experience of severe abuse [17]. Thus, social desirability may contribute to the findings revealing that males perpetrate less dating violence than females [3,8,16]. Another problem concerning studies of dating violence prevalence is that there is evidence that many adolescents and young adults normalize violent behaviors within the relationship, do not consider themselves victims, and are not afraid of such violence [18,19].

Research has reported that dating violence is associated with an array of adverse physical and mental outcomes, including emotional and psychological problems for both victims and perpetrators [20,21]. In addition to potential physical harm and injury, physical, psychological, and sexual dating violence victimization has been consistently associated with many adverse outcomes, including mental symptoms, low self-esteem, poorer well-being, risk for future victimization, and increased substance use [21,22,23,24]. In longitudinal studies, dating violence victimization has also been found to be related to many adverse outcomes, including depressive symptoms, anxiety, poorer well-being, suicidal ideation, and increased revictimization [21,23,24]; however, the associations are not consistent across studies when considering the type of dating violence experienced and the gender of the victim [24]. In a longitudinal study by Exner-Cortens et al. [23] conducted with a nationally representative sample of US middle and high schools, it was found that female students experiencing dating victimization, as compared with participants reporting no dating violence victimization, reported increased IPV victimization, depressive symptoms, suicidal ideation, smoking, and heavy episodic drinking 5 years later. Male participants experiencing dating victimization reported increased suicidal ideation, antisocial behavior, marijuana use, and IPV victimization 5 years later. Dating violence has also been found to be an important risk factor for adult partner violence [25,26,27,28], with evidence that many battered women had begun the relationship with the abuser before the age of 29 years [27].

A great number of studies have focused on understanding the risk factors associated with IPV, and numerous risk factors have been found, although findings across studies are often contradictory [29]. According to the Centers for Disease Control and Prevention (CDC) [30], a combination of individual, relational, community, and social factors enhances the risk of becoming a perpetrator of IPV. Although they are influencing factors, they may not be causes. Many individual factors have been cited, including (but not limited to) demographic factors such as low education, young age (adolescence and young adulthood), and low income or unemployment. Other individual risks involve low self-esteem, anger and hostility, depression and suicide attempts, delinquent or aggressive behavior as a youth, deficits in conflict resolution and problem-solving skills, heavy alcohol and drug use, antisocial personality traits, impulsivity, alexithymia, stress, history of physical or emotional abuse or neglect in childhood, emotional dependence and insecurity, traditional gender role beliefs, attitudes accepting or justifying aggression and violence, and attitudes about acceptability and expected consequences of dating violence [3,10,13,29,30,31,32,33,34,35,36,37,38,39]. Relational factors cover relationship conflicts such as possessiveness, tension, or jealousy; desire for control of the relationship; association with aggressive and antisocial peers; and childhood history factors including witnessing interparental partner violence, experiencing poor parenting, and experiencing child abuse and neglect [3,10,13,29,30,31,32,33,34]. Community factors include communities with high rates of poverty, unemployment, and limited economic and educational opportunities; communities with high rates of crime and violence, with easy access to drugs and alcohol; and communities that sanction IPV poorly [10,30,31]. Finally, social factors consider traditional gender norms and gender inequality, cultural norms that support violence and aggression, social inequalities, and weak educational, social, health, and economic policies or laws [10,30,39].

Although a large body of research analysing dating violence has been conducted in various countries, most has focused on both adolescents and college students, with little research centering specifically on emerging adulthood, a period of life in which rates of dating violence are very high and are associated with negative consequences [40]. According to Breiding et al. [41], 47.1% of women and 38.6% of men IPV victims were between 18 and 24 years old when they first experienced violence by an intimate partner.

Emerging adulthood generally refers to the ages between 18 and 29 years and is considered a new life stage between puberty-based adolescence and a more stable young adulthood [42]. It is a distinct period in its subjective and demographic dimensions, and that offers the highest opportunity for exploring identity in the areas of worldviews, love, and work [43]. According to Arnett [44], emerging adulthood involves five main features: (1) identity exploration, a time when young people probe feasible life options, especially in love and at work; (2) instability in love, place of residence, and in work; (3) self-focus; (4) feelings in between, neither adult nor adolescent, in transition; and (5) possibilities/optimism. Emerging adulthood is “the age of possibilities, when many different futures remain possible, when little about a person’s direction in life has been decided for certain. It tends to be an age of high hopes and great expectations” [42] (p. 15) as this stage offers “the potential for changing dramatically the direction of one’s life” (p. 16).

All of this makes emerging adulthood a particularly relevant period of the life cycle for the study of dating violence. Given that this is a period characterized by exploration and change in romantic relationships, this paper analyzes dating violence by performing a differential analysis focused on gender and on whether women and men currently have a partner. Moreover, although mental health has been traditionally defined as the absence of psychopathologies such as anxiety or depression [45], this conceptualization has been shown to be insufficient from a perspective on lifespan development, since mental health has been defined “as a positive phenomenon that is more than the absence of mental illness” [45] (p. 110). Therefore, in addition to analysing common mental symptoms, this study addresses life satisfaction, which is considered to be the cognitive component of subjective well-being [46] and self-esteem. The main aim of the present work is to expand knowledge of dating violence in emerging adulthood, probing into the relevance of gender and of having or not having a current partner in the victimization and perpetration of such violence. Furthermore, the association of such violence with mental symptoms and life satisfaction is analyzed too. The third aim of the study is to test the relevance of the traditional gender role attitudes and the internalization of characteristics traditionally associated with feminine/expressive and masculine/instrumental traits in dating violence victimization and perpetration in emerging adulthood.

## 2. Materials and Methods

### 2.1. Participants

The sample included 930 volunteer participants (55.4% women and 44.6% men) from the Spanish general population aged between 18 and 29 years. Table 1 displays the socio-demographic features of the groups of women and men. As seen, over half of the sample were students, nearly one-fourth were employees, and almost 12% were unemployed, percentages that were not significantly different in women and men, χ^2^(1, *N* = 918) = 1.87, *p* = 0.392. Most frequently, they had completed a high school degree or professional training, which was found in 63.6% of the sample. In women, 23.7% had completed university education, and 11.4% had completed elementary education; in men, the percentages were 17.1% and 21%, respectively. The differences between women and men in educational level were statistically significant, χ^2^(2, *N* = 852) = 16.87, *p* < 0.001. All participants had either at the time or previously a romantic partner, although they had not married or were not currently living with a partner. At the time of completing the tests, 58.3% of the women’s sample and 57.1% of the men’s sample had a partner, while 41.7% of women and 42.9% of men had no partner, the differences in percentage not being statistically significant, χ^2^(1, *N* = 930) = 0.12, *p* = 0.726. Women and men did not either differ in age, as seen in Table 1.

### 2.2. Procedure

Access to the sample was through educational, work, and community association centers located across different Spanish localities, as well as through the social net of psychology undergraduates and graduate students who participated in the data collection, after being trained to do so, and received course credit for their participation. All the evaluation instruments as well as the sociodemographic data gathering sheet were self-completed manually and individually on a paper form. This form did not include the name or any other data that could identify the participants.

The sample used in this study was selected from a larger study on gender, violence and health using the following criteria: (1) Participants were between 18 and 29 years old. (2) Previous or current dating relationship with a person of different sex but neither married nor living currently with a partner. (3) Completion of all questionnaires and scales.

The study was conducted in accordance with the Declaration of Helsinki and its later amendments. All participants gave their verbal informed consent before completing the questionnaires and scales and had the possibility to drop out of the study at any time. Written informed consent was waived due to the fact that informed consent was sought verbally; to fully respect anonymity, we do not ask them to sign the informed consent since it is not necessary and such a signature implies a threat to their anonymity. The present study constitutes part of a research on gender, violence, and health that was approved by the Ethics Committee on Animal Research and Welfare of the University of La Laguna (ethical approval code 2013-0058).

### 2.3. Instruments

Violence was measured using a 42-item scale consisting of two parts containing 21 items each: (a) violence victimization, which collected information on the frequency with which she/he had been a victim of violent or controlling behaviors by his/her romantic partner, and (b) violence perpetration, which asked about the frequency with which she/he had perpetrated such violence towards his/her romantic partner. The scale also collects information on the sex of the romantic partner whose violence is being assessed. The victimization and perpetration subscales are structured into three violence types: (1) psychological violence, which consists of 9 items describing behaviors such as insults, humiliation, manipulation, belittling, blaming, or control; (2) physical violence, which included 10 items describing behaviors such as pushing, slapping, hitting, beating, or threats of aggression, and (3) sexual violence, which comprises two items, one referring to imposing undesired or disliked sexual behaviors by force or threats; and the other referring to break-up threats if not complying with the romantic partner’s sexual desires. The response format was a three-point Likert-type scale: never, scored with 0; sometimes, scored with 1; and many times, scored with 2, higher scores indicating a greater level of psychological, physical, and sexual violence. The possible range of responses allowed by the scale, for both victimization and perpetration, was between 0 and 42 for total violence, 0 and 18 for psychological violence, 0 and 20 for physical violence, and between 0 and 4 for sexual violence.

In this study, the Cronbach’s alpha of 21 items assessing total victimization had a value of 0.86 and 0.85 for 21 items rating total perpetration. The Cronbach’s alpha for the different types of violence was 0.82 for psychological violence victimization, 0.78 for physical violence victimization, and 0.63 for sexual violence victimization; 0.69 for psychological violence perpetration, 0.85 for physical violence perpetration, and 0.52 for sexual violence perpetration.

The Spanish version [47] of the Goldberg general health questionnaire in a scaled version (GHQ-28) [48] was used. The GHQ-28 is a self-administered tool designed to detect current mental disturbances that is widely used as a measure of common mental disorders in public health surveys [49] and features as one of the most commonly used mental health assessment tools [50]. The GHQ-28 describes individual health status in terms of four non-independent dimensions. Each dimension consisted of 7 items: somatic symptoms, anxiety and insomnia, social dysfunction, and severe depression. Items were scored following the Likert-type procedure that assigns a weight to each score, ranging from 0 to 3, with higher scores indicating greater somatic, anxiety and insomnia, social dysfunction, and severe depression symptoms. For the current sample, the Cronbach’s α for each scale was, respectively, 0.82, 0.89, 0.79, and 0.88.

The Spanish version of satisfaction with life scale (SWLS) [51] was used. The SWLS assesses a respondent’s overall judgment of his or her satisfaction with life. SWLS analyses an individual’s conscious evaluative judgement of her or his life on the basis of their own criteria [49]. The scale consists of five items rated on a 7-point Likert-type scale, with higher scores indicating greater life satisfaction. For the present sample, the Cronbach’s *α* coefficient was 0.85.

The Spanish version of Rosenberg self-esteem scale (RSES) [52], adapted by Martín-Albo et al. [53], was used. The RSES consists of ten items and measures global self-esteem. It is a scale widely used to assess self-esteem, and its psychometric features have been studied worldwide [54]. Participants were asked to rate each item on a 4-point scale, and higher scores indicated higher self-esteem. In this study, Cronbach’s α was 0.87.

A gender roles attitudes questionnaire (ARG) was used [55]. It is a self-report questionnaire consisting of 22 items that assess traditional attitudes toward familial and social roles for women and men. The participants were asked to state how much they agree or disagree with each statement on a seven-point Likert-type scale, with higher scores reflecting more traditional gender role attitudes. For the present sample, the Cronbach’s α coefficient was 0.88.

The short form of Bem Sex Role Inventory (BSRI) [56] was used. The BSRI is a self-report inventory that assesses the degree to which a person self-attributes socially desirable personality characteristics that are stereotypically associated with women and men. It consists of 20 short sentences or adjectives and is structured in two scales, each composed of 10 items: the feminine/expressive scale that includes characteristics traditionally considered as feminine, such as being compassionate, affectionate, kind, tender, understanding, and sensitive to the needs of others; and the masculine/instrumental scale that includes characteristics and traits traditionally considered as masculine, such as being independent, competitive, assertive, dominant, and aggressive. All items were translated (with back-translation) by two bilingual Spanish–English speakers residing in the United States: a professional translator whose native language is English and a researcher whose native language is Spanish. People’s endorsement of the attributes is rated on a 7-point Likert scale, with higher scores indicating a greater level of expressiveness or instrumentality. For the present sample, the Cronbach’s α for the masculine/instrumental scale had a value of 0.76 and 0.86 for the feminine/expressive scale.

Sociodemographic data were collected by using a sheet asking about gender, (men, women, other) age, educational level, marital status, whether or not they had a partner, and occupation. Only people who self-identified as women or as men were included in the current study.

### 2.4. Statistical Analyses

Statistical analyses were conducted with SPSS 22.0 (IBM Corporation, Armonk, NY, USA) software. Internal consistency was measured by using Cronbach’s alpha coefficient. Descriptive analyses were computed to determine the frequency with which the different violent behaviors occurred and to describe the sociodemographic characteristics of the participants. Comparisons between women and men in the demographic variables were performed using Pearson’s chi-square, except in the case of age, which was calculated using Student’s *t*-test, since it was a quantitative variable. Differences between women and men and between people with a current partner and with a former partner in dating violence victimization and perpetration were obtained by performing six analyses of variance between subjects (ANOVA). In each ANOVA, the independent variables were gender (women and men) and having or not having a current partner (current partner, former partner), while the dependent variables were victimization by psychological, physical, and sexual violence and perpetration of physical, psychological, and sexual violence. The bivariate associations between victimization and perpetration of dating violence were computed using Pearson’s r correlation coefficient, with the exception of educational level, in which Spearman’s Rho was employed, since it is an ordinal variable. To achieve the third study aim, hierarchical multiple regression analyses were conducted. Logarithmic transformations were used on violence victimization and perpetration to reduce skewness, following the recommendations of Barbara G. Tabachnick and Linda S. Fidell [57]. In each regression analysis, age was included in the first step (model 1) to control their effect. In the second step (model 2), traditional gender role attitudes and self-identification with feminine/expressive and masculine/instrumental traits scores were introduced. In the third step (model 3), the dating violence perpetration score was entered when the criterion variable was the score for dating violence victimization, whereas the dating violence victimization score was included when the criterion variable was the score for dating violence perpetration. For all analyses, *p* values less than 0.05 were considered statistically significant.

## 3. Results

### 3.1. Descriptive Analyses and Comparison in Victimization and Perpetration of Violence

Of the total sample, 63.5% reported having been victims of dating violence; 59.1% experienced psychological victimization, 20.9% experienced physical violence, and 7.5% had experienced sexual victimization. Men with former partner cited the highest frequency of having been victims of dating violence (77.5%), followed by women with former partner (63.3%), men with current partner (66.2%), and women with current partner (53.3%). A total of 57.6% of the sample reported having perpetrated dating violence, with 53.8% reporting psychological violence, 14.8% physical violence, and 3.2% sexual violence. The perpetration of dating violence was more frequent in men with a former partner (60.7%), followed by men with a current partner (58.2%), women with a current partner (57.6%), and women with a former partner (56.3%).

Table 2 shows the range of responses of the four groups tested as regards dating violence victimization and perpetration of such violence. As seen in Table 2, the maximum score for total victimization reported by the four groups is well below the maximum range allowed by the scale. The same holds true for total dating violence perpetration, except in the group of men with a former partner, whose maximum score for total perpetration was 36.

Table 3 displays the main results of two-factor ANOVAs with participants’ gender (women and men) and having or not having a current partner (current partner, former partner) as between-subject factors and dating violence victimization as the dependent variable; Table 4 displays the main results with dating violence perpetration as the dependent variable. As seen in both tables, no statistically significant interaction exists between gender × having or not having a current partner. When victimization by dating psychological violence was considered as the dependent variable, the main effects of gender and of having or not having a partner were statistically significant, although the effect size was larger for the main effects of having or not having a partner. As seen in Table 3, men reported greater victimization by psychological dating violence than women, although the effect size was very small. In addition, women and men without a current partner reported more psychological violence victimization than women and men with a current partner. When the dependent variable was physical violence victimization, only the main effect of gender was statistically significant, with men reporting more physical violence victimization than women; however, the effect size was small. When the dependent variable was sexual violence victimization, only the main effects of having or not having a partner were statistically significant and proved that sexual victimization in women and men with no current partner was greater than in those who currently have a partner; once again, the effect size was small.

When the perpetration of dating violence was considered the dependent variable, only the principal effect of gender on physical violence perpetration was statistically significant. Although the effect size was very small, men reported more physical dating violence perpetration than women (see Table 4).

### 3.2. Correlations between Study Variables

Table 5 displays the correlation coefficients between the three forms of dating violence victimization and between the three forms of dating violence perpetration for the four groups. As seen, the association of victimization by psychological violence with victimization by physical violence is constant in all those four groups, and the effect size of the association is moderate. Except for the men’s group with a current partner, psychological violence victimization was also associated with sexual violence victimization, with its effect size being small. In addition, physical violence victimization was associated in both men’s groups with sexual violence victimization, and the effect size was small. Psychological violence perpetration was associated with physical violence perpetration; the effect size for either the women’s and the men’s groups with a current partner was moderate and small for the men’s and women’s groups with a former partner. In addition, in the men’s group with a current partner, sexual violence perpetration was associated with physical violence perpetration, and its effect size was moderate, whereas the effect size turned small in the case of psychological violence perpetration.

Table 6 displays the correlation coefficients between violence victimization and perpetration for the four groups. As seen, in all groups, both total victimization and victimization by different violence types were statistically significant when associated with perpetration of the same type of violence, although the effect size varied among the different groups. The strongest association was between victimization and perpetration of physical violence in the men’s group with a partner, where the effect size of the association was large, whereas the smallest association occurred between victimization and perpetration of sexual violence in the men’s group without a partner, where the effect size was small. In the women’s group without a partner, the associations between victimization and perpetration of total, psychological, and physical violence were lower than in the other groups, whereas the men’s group with a partner had the highest associations between total, physical, and sexual violence. Furthermore, in all groups except the men’s group with a current partner, the lowest association between victimization and perpetration was for sexual violence.

Table 7 displays the correlation coefficients between dating violence victimization and dating violence perpetration with the study’s variables for the women’s groups and Table 8 for the men’s groups. As seen, all the groups indicate that educational level is independent of dating violence victimization and perpetration, as is age, except in men with a former partner, where older age is associated with greater psychological and total victimization, although the effect size is small. The results of the correlations between victimization by dating violence and mental symptomatology, life satisfaction, and self-esteem show the existence of important differences between the four groups. The greatest associations were found in women without a current partner and in men with a current partner, whose greater violence victimization was associated with greater anxiety and insomnia, somatic and severe depression symptoms, lower life satisfaction, and lower self-esteem. In women with a current partner, greater psychological and total violence victimization was associated with more anxiety and insomnia symptoms and lower life satisfaction, whereas in men without a current partner, there was no statistically significant correlation between dating violence victimization and their mental symptomatology and life satisfaction, except between sexual victimization and self-esteem, as men with lower self-esteem presented higher sexual victimization.

In men with a current partner, perpetration of dating violence was also associated with greater mental symptoms, lower life satisfaction, and lower self-esteem. Although the effect size was low or very low, in women with a current partner, perpetration of dating violence was associated with more symptoms of anxiety and insomnia, with greater social dysfunction, and with lower self-esteem and life satisfaction; in the case of women without a current partner, perpetration of psychological and total violence was associated with more somatic, anxiety and insomnia, and severe depression symptoms. In men without a current partner, there were only two statistically significant correlations, with more psychological violence perpetration associated with more somatic symptoms and more total violence perpetration associated with lower self-esteem.

The masculine/instrumental trait was independent of dating violence victimization and perpetration except for men without a current partner, since greater psychological and total victimization were associated with greater internalization of the characteristics of the masculine/instrumental trait. In men and women with a current partner, higher scores on the feminine/expressive trait were associated with lower dating violence victimization and perpetration; as for men without a current partner, the feminine/expressive trait correlated with physical violence victimization and with physical and total violence perpetration, with less violence with higher scores on the feminine/expressive trait.

In all groups, except for women without a current partner, statistically significant correlations were found between traditional gender role attitudes and dating violence victimization and perpetration, although the association was stronger in men without a current partner, with more psychological, physical, sexual and total violence being perpetrated by men with more traditional gender role attitudes, who also reported more victimization by all types of violence except for sexual violence.

Additionally, in women and men with a current partner, more traditional gender role attitudes were associated with greater victimization and perpetration of psychological, physical and total violence, although in the men’s group, the correlation coefficient between traditional gender role attitudes and physical violence victimization was not statistically significant.

### 3.3. Risk and Protective Factors of Dating Violence Victimization and Perpetration

Table 9 displays the summary of the hierarchical multiple regression results predicting dating violence victimization for the women’s and men’s groups and Table 10 for the prediction of dating violence perpetration. As seen in Table 9, in the four groups, the most important variable in predicting greater dating violence victimization in the final model (model 3) is greater perpetration of dating violence. The regression equation of this model included, in addition to the violence perpetration score, scores on masculine/instrumental and feminine/expressive traits, traditional gender role attitudes, and age. In this final model, the group of women with a current partner proved that more traditional gender role attitudes also predicted greater dating violence victimization. The adjusted *R*^2^ score of 0.47 indicated that 47% of the total variance in dating violence victimization was explained in this group. In the final model too, the women with a former partner group revealed that a lower score on the masculine/instrumental trait was also a statistically significant predictor of higher dating violence victimization, with a total percentage of variance explained of 30%; yet in the case of men with a current partner, the only statistically significant predictor of dating violence victimization was the perpetration of such violence, the percentage of variance explained being 32.4%, while in men with a former partner, in addition to greater dating violence perpetration, greater age predicted greater dating violence victimization, the total percentage of variance explained being 39.1%. Although in both men’s groups, more traditional gender role attitudes were a statistically significant predictor of greater dating violence victimization in model 2, this variable was no longer statistically significant when dating violence perpetration was included in the regression equation in model 3.

In the final model, the main predictor of greater dating violence perpetration in all those four groups was greater violence victimization by such a partner. In the two women’s groups and in the men’s group with a current partner, dating violence perpetration was also predicted by lower scores on the feminine/expressive trait. In addition, in both men’s groups, more traditional gender role attitudes predicted greater dating violence perpetration. The percentages of variance explained were 52.7% in men with a current partner, 41.4% in women with a current partner, 37.4% in men with a former partner, and 23.5% in women with a former partner.

## 4. Discussion

The main aim of the present work was to expand knowledge of dating violence in emerging adulthood by analysing the relevance of gender and having or not having a current partner in the victimization and perpetration of such violence, as well as in the association between dating violence and victims’ and perpetrators’ mental health. The results show that dating violence victimization is frequent in emerging adulthood, affecting more than half of the sample and being reported more frequently by men and women with a former partner than by men and women with a current partner. The percentages were within the range of victimization for dating violence found in other studies [1,12,40]. The most prevalent violence type was psychological violence, which was reported by slightly more than half of the sample, one-fifth reported physical violence, and less than 10% reported having been victims of dating sexual violence. These results are consistent with the overall hierarchy of violence found across almost all investigations, in which psychological/emotional violence is most prevalent and sexual violence least prevalent [58,59]. In the present study, the prevalence of dating violence perpetration was slightly lower than the prevalence of dating violence victimization, with dating violence perpetration proved to be quite similar in women and men and in people with a current partner and with a former partner. Prevalence perpetration rates ranged from 60.7% in men with former partner to 56.3% in women with former partner. These results converge with those of other investigations in which no gender differences in the perpetration of psychological violence have been found [60]. The results of the current study also converge with research from other countries conducted with emerging adults where the majority of the sample has been found to have experienced or perpetrated some type of dating violence [40].

When analysing mean gender differences in dating violence, it was found that men reported greater victimization by psychological and physical violence and greater perpetration of physical violence than women. These data converge with those of other studies where it has been found that men report greater victimization by physical and psychological violence than women [8]. In any case, it should be noted that in the present work, the effect size was low for physical violence victimization and very low or even negligible for psychological violence victimization and physical violence perpetration. The most frequent forms of victimization by physical violence were small wounds, slapping, and pushing, with throwing objects and threats of physical violence being less frequent. Furthermore, only two people reported having been victims of more serious violence, such as beatings, or tried to hurt by choking or suffocating: a man with a current partner and a woman with a former partner. Only three participants had suffered significant injuries from dating violence: two women, one with a current partner and one with a former partner, and one man with a former partner. Moreover, only men reported perpetrating any severe violence. Therefore, these data are consistent with those of other studies where it has been shown that men are more likely to inflict injuries [8,16], whereas it is more common for women to suffer from injuries [8]. Additionally, we found that women and men with a former partner reported more victimization by sexual and psychological violence than women and men with a current partner. Although the reasons why people with a current partner report less psychological and sexual dating violence victimization than those with a former partner are unknown, we hypothesize that the relationship did not continue as a consequence of the violence suffered, a hypothesis that should be tested in future research. It is also noteworthy that the effect size of the differences in victimization by psychological violence was four times larger for the main effect of having a former or a current partner than for the main effect of gender.

The current study has concluded that in all groups, violence victimization and perpetration were associated, with most effect sizes being medium. These results are consistent with previous research and reinforce what has been argued: that violence in dating relationships is bidirectional and that men and women perpetrate and experience different types of violence [14], although this is not always the case. It should be noted that the results of the current study suggest that in dating violence victimization and in the perpetration of physical violence, gender and whether it is a current or former intimate partner relationship matters, something that should be examined in future research.

Dating violence victimization and perpetration were independent of educational level and were only associated with age in the men’s group with former partner. Therefore, the results of the current study do not support that there is a higher risk of dating violence perpetration in people with low educational attainment, as has been proposed in the previous literature [10], although it should be noted that the sample of the current study is limited to persons between 18 and 29 years of age residing in Spain, so we may speculate on the possibility that in other age ranges and/or in other countries a lower level of education is a risk factor for intimate partner violence.

Analysis of associations between dating violence victimization and perpetration with mental health shows that greater total and psychological violence victimization are associated with greater anxiety and insomnia symptoms and with lower life satisfaction in all groups except in men with a former partner. In addition, perpetration of such violence was also paired up with greater anxiety and insomnia symptoms and lower life satisfaction in women and men with a current partner. The results of the current study show that the association patterns between dating violence victimization and perpetration with mental health depend on gender and on whether there is a current or a former partner. Although the reasons for such differences are unknown, gender socialization and gender norms could be explanatory factors for the differences in the association between dating violence and mental health observed in the four groups. Thus, the impact could be greater in men with a current partner because being victims of women’s violence goes against gender stereotypes and norms that place men at the top of the social hierarchy [61], attribute greater value to men and masculinity [62], and associate values such as strength, assertiveness, dominance, independence, achievement, or aggressiveness [56]. Therefore, being a victim of violence by a woman who is his current partner could cause him to question his worth as a man and generate mental symptoms, lower self-esteem, and less life satisfaction. Such factors may not be relevant in men who do not have a current partner because, as they are no longer in this partner relationship, there is no victimization that challenges all these values and norms. In addition, gender trait stereotypes consider that men should be independent, dominant, willing to take risks, and determined in their positions [56], so what happened with a previous partner would not influence their current mental health nor would influence their self-esteem. In contrast, in women lacking a current partner, the impact of violence by a former partner may persist even if the partner relationship does not continue because, according to socialization and traditional gender norms, women should be sensitive to the needs of others, compassionate, affectionate, understanding, and sympathetic [56], so it could be that women feel, at least partially, responsible for the violence suffered and/or attribute greater importance to it, which is why victimization by a former partner would be associated with mental symptoms, lower self-esteem, and less life satisfaction. The lower association between victimization and mental health in women with a current partner could be due to two factors: the first is that they are the group that reported less dating violence victimization, which could explain the lower impact that dating violence seems to have on women’s mental health; the other is that they could attribute less relevance to such violence, since there is evidence that women being in a partner relationship rate intimate partner violence as less serious [63]. These are hypotheses that should be tested in future research.

The third aim of the study was to test the relevance of the traditional gender role attitudes and the internalization of characteristics traditionally associated with feminine/expressive and masculine/instrumental traits in the victimization and perpetration of dating violence in emerging adults. Regression analyses show that in model 2, which includes scores on traditional gender role attitudes and feminine/expressive and masculine/instrumental traits, it is found that more traditional gender role attitudes predicted greater dating violence victimization in all groups, except in the group of women with a previous partner; however, this variable is no longer statistically significant in the two men’s groups when dating violence perpetration scores are included in the regression equation, which are the most important predictors in all four groups, with greater dating violence victimization in persons with greater dating violence perpetration. As regards the prediction of greater dating violence perpetration, it can be stated that, in addition to greater victimization by such violence, the fact of less internalization of the characteristics associated with the feminine/expressive trait such as solidarity, understanding, warmth, sensitivity to the needs of others, or willingness to comfort others, was also a statistically significant predictor in all groups, except for the group of men with a former partner. Additionally, in both men’s groups, greater dating violence perpetration was related with more traditional gender role attitudes. These data are consistent with those of other studies that have shown the relevance of sexism, gender norms, and traditional gender attitudes in dating violence [12,29,36,37,39].

### Limitation

This study has a number of limitations, including that all the data were obtained through self-reports, which can be an important source of bias, especially social desirability. A second limitation is that this is a cross-sectional study, so causal assumptions cannot be made. In addition, the sample is not random, so the sample could be subject to biases such as selection bias. Finally, another limitation of the present study is that the internal consistency of the subscales assessing victimization and perpetration of sexual violence is questionable, perhaps as a consequence of the fact that such scales only contain two items for the measurement of sexual violence.

## 5. Conclusions

Despite limitations, the results of the present study allow us to conclude that dating violence is a significant problem in emerging adulthood, being a complex phenomenon whose nature and effects seem to be considerably determined by gender and by having former or current partner. Additionally, dating violence is a threat to the mental health of people at this important life cycle stage. Another important reason for prevention and intervention in dating violence is that there is evidence that intimate partner violence tends to start early in many relationships, as there is documented proof that many battered women had begun the relationship with the abuser before the age of 29. The results of the present work may be useful for the design of strategies, policies, and programs aimed at the prevention of dating violence. The results also indicate the importance of interventions with victims and perpetrators of dating violence, given that the main predictor of dating violence perpetration is dating violence victimization and the main predictor of dating violence victimization is dating violence perpetration.

## Figures and Tables

**Table 1:** Demographic characteristics of the groups of women and men.

	Women (*n* = 515)		Men (*n* = 415)		χ^2^-Value
	*n*	%	*n*	%	
Occupation					
Employed	122	23.9	111	27.3	1.87
Unemployed	58	11.4	50	12.3	
Student	331	64.8	246	60.4	
No data	4		8		
Educational level					
Elementary studies	56	11.4	76	21.0	16.87 ***
High school degree or professional training	318	64.9	224	61.9	
University degree	116	23.7	62	17.1	
No data	25		53		
Marital status					
Never married unpartnered	215	41.7	178	42.9	0.12
Never married with partner	300	58.3	237	57.1	
	***M***	***SD***	***M***	***SD***	***t*** **value**
Age	22.26	2.59	22.59	2.59	−1.79

Note: *** *p* < 0.001.

**Table 2:** Range of violence victimization and perpetration scores for the four groups.

Variable	Men		Women		Total
	Current Partner	Former Partner	Current Partner	Former Partner	
**Victimization**					
Total	0–27	0–23	0–25	0–23	0–27
Psychological	0–16	0–9	0–15	0–13	0–16
Physical	0–9	0–12	0–14	0–9	0–14
Sexual	0–4	0–3	0–3	0–2	0–4
**Perpetration**					
Total	0–12	0–36	0–9	0–18	0–36
Psychological	0–12	0–12	0–9	0–10	0–12
Physical	0–10	0–20	0–5	0–8	0–20
Sexual	0–2	0–4	0–2	0–2	0–4

**Table 3:** Means (*M*), standard deviations (*SD*), and two-way ANOVA statistics for dating violence victimization as dependent variable.

Variable	Former Partner		Current Partner		ANOVA		
	*M*	*SD*	*M*	*SD*	Effect	*F* Ratio	η_p_^2^
Psychological violence							
Men	2.71	3.08	1.81	2.18	Gender	5.26 *	0.006
Women	2.29	3.28	1.42	2.18	Partner	24.95 ***	0.026
Interaction gender × partner					G × P	0.00	0.000
Physical violence							
Men	0.75	1.61	0.63	1.47	Gender	17.56 ***	0.019
Women	0.39	1.31	0.26	0.91	Partner	1.85	0.002
Interaction gender × partner					G × P	0.00	0.000
Sexual violence							
Men	0.20	0.64	0.09	0.42	Gender	0.28	0.000
Women	0.21	0.57	0.05	0.28	Partner	17.82 ***	0.019
Interaction gender × partner					G × P	0.87	0.001

Notes: G × P = interaction gender × partner; * *p* < 0.05; *** *p* < 0.001.

**Table 4:** Means (*M*), standard deviations (*SD*), and two-way ANOVA statistics for dating violence perpetration as dependent variable.

Variable	Former Partner		Current Partner		ANOVA		
	*M*	*SD*	*M*	*SD*	Effect	*F* Ratio	η_p_^2^
Psychological violence							
Men	1.23	1.67	1.20	1.66	Gender	0.17	0.000
Women	1.11	1.55	1.23	1.69	Partner	0.14	0.000
Interaction gender × partner					G × P	0.45	0.000
Physical violence							
Men	0.40	1.23	0.39	1.60	Gender	4.59 *	0.005
Women	0.17	0.63	0.29	0.92	Partner	0.47	0.001
Interaction gender × partner					G × P	0.81	0.001
Sexual violence							
Men	0.06	0.30	0.08	0.42	Gender	1.87	0.002
Women	0.05	0.30	0.04	0.25	Partner	0.04	0.000
Interaction gender × partner					G × P	0.43	0.000

Notes: G × P = interaction gender × partner. * *p* < 0.05.

**Table 5:** Intercorrelations between violence types for the four groups.

	Women with Current Partner		Women with Former Partner		Men with Current Partner		Men with Former Partner	
	Physical	Sexual	Physical	Sexual	Physical	Sexual	Physical	Sexual
**Violence victimization**								
Psychological	0.59 ***	0.15 *	0.52 ***	0.28 ***	0.40 ***	0.08	0.49 ***	0.29 ***
Physical		0.02		0.09		0.23 ***		0.18 *
**Violence perpetration**								
Psychological	0.43 ***	−0.03	0.20 **	0.12	0.51 ***	0.20 **	0.20 **	−0.01
Physical		−0.03		0.01		0.48 ***		0.07

Notes: Statistically significant correlation coefficients are shown in bold; * *p* < 0.05; ** *p* < 0.01; *** *p* < 0.001.

**Table 6:** Correlations between violence victimization and perpetration for women’s and men’s groups.

	Total Violence	Psychological Violence	Physical Violence	Sexual Violence
**Women with a partner**				
Total violence	0.68 ***			
Psychological violence		0.66 ***		
Physical violence			0.61 ***	
Sexual violence				0.36 ***
**Women without a partner**				
Total	0.46 ***			
Psychological		0.39 ***		
Physical			0.41 ***	
Sexual				0.38 ***
**Men with a partner**				
Total	0.75 ***			
Psychological		0.62 ***		
Physical			0.80 ***	
Sexual				0.73 ***
**Men without a partner**				
Total	0.60 ***			
Psychological		0.51 ***		
Physical			0.66 ***	
Sexual				0.26 **

Notes: Statistically significant correlation coefficients are shown in bold; ** *p* < 0.01; *** *p* < 0.001.

**Table 7:** Correlations between dating violence and study variables for the women’s groups.

	Total Victimization	Psychological Victimization	Physical Victimization	Sexual Victimization	Total Perpetration	Psychological Perpetration	Physical Perpetration	Sexual Perpetration
**Women with current partner**								
Age	−0.07	−0.05	−0.10	0.00	0.02	0.04	−0.03	0.04
Educational level ^a^	−0.09	−0.11	−0.11	−0.05	−0.02	−0.02	−0.02	−0.04
Somatic symptoms	0.01	0.02	−0.01	0.00	0.05	0.05	0.04	−0.02
Anxiety and insomnia symptoms	0.15 **	0.15 **	0.09	0.07	0.15 **	0.13 **	0.12 *	0.02
Social dysfunction	0.10	0.11	0.04	0.09	0.14 *	0.13 *	0.08	0.06
Severe depression symptoms	0.04	0.04	0.03	−0.04	0.11	0.08	0.11	0.02
Life satisfaction	−0.14 *	−0.13 *	−0.09	−0.09	−0.19 **	−0.16 **	−0.17 **	0.01
Self-esteem	−0.08	−0.07	−0.07	−0.04	−0.18 **	−0.15 **	−0.15 **	−0.02
Masculine/instrumental trait	0.01	0.02	−0.02	−0.02	0.02	0.00	−0.05	0.02
Feminine/expressive trait	−0.12 *	−0.13 *	−0.07	−0.02	−0.23 ***	−0.20 ***	−0.17 **	−0.03
Traditional gender role attitudes	0.21 ***	0.19 **	0.18 ***	0.08	0.17 **	0.15 **	0.13 *	0.02
**Women with former partner**								
Age	−0.03	−0.03	0.01	−0.08	−0.07	−0.05	−0.08	0.01
Educational level ^a^	0.03	0.05	−0.05	−0.10	0.08	0.08	−0.08	0.06
Somatic symptoms	0.29 ***	0.26 ***	0.24 ***	0.09	0.24 **	0.21 **	0.13	0.10
Anxiety and insomnia symptoms	0.31 ***	0.30 ***	0.22 **	0.09	0.24 ***	0.21 **	0.11	0.13
Social dysfunction	0.15 *	0.13	0.11	0.13	0.05	0.05	−0.03	0.01
Severe depression symptoms	0.21 **	0.17 *	0.18 **	0.20 **	0.16 *	0.12	0.12	0.08
Life satisfaction	−0.19 **	−0.17 *	−0.17 *	−0.04	−0.09	−0.09	−0.02	−0.07
Self-esteem	−0.17 *	−0.14 *	−0.13	−0.18 **	−0.07	−0.05	−0.05	−0.06
Masculine/instrumental trait	−0.13	−0.13	−0.08	−0.07	−0.01	0.00	0.02	−0.05
Feminine/expressive trait	0.05	0.05	0.06	−0.08	−0.07	−0.12	0.10	−0.04
Traditional gender role attitudes	−0.05	−0.04	−0.06	−0.01	0.05	0.06	−0.02	0.08

Notes: ^a^ = Correlation coefficient calculated with Spearman’s *Rho*; statistically significant correlation coefficients are shown in bold. * *p* < 0.05; ** *p* < 0.01; *** *p* < 0.001.

**Table 8:** Correlations between dating violence and study variables for the men’s groups.

	Total Victimization	Psychological Victimization	Physical Victimization	Sexual Victimization	Total Perpetration	Psychological Perpetration	Physical Perpetration	Sexual Perpetration
**Men with current partner**								
Age	−0.02	0.04	−0.10	−0.01	−0.02	0.03	−0.06	−0.03
Educational level ^a^	−0.04	−0.06	−0.06	0.06	−0.07	−0.10	−0.01	0.05
Somatic symptoms	0.22 **	0.17 **	0.16 *	0.16 *	0.20 **	0.19 **	0.18 **	−0.01
Anxiety and insomnia symptoms	0.22 **	0.18 **	0.19 **	0.07	0.20 **	0.22 **	0.17 **	−0.03
Social dysfunction	0.13 *	0.14 *	0.03	0.14 *	0.14 *	0.14 *	0.10	0.05
Severe depression symptoms	0.27 ***	0.19 **	0.24 ***	0.26 ***	0.34 ***	0.32 ***	0.29 ***	0.12
Life satisfaction	−0.28 ***	−0.27 ***	−0.18 **	−0.12	−0.24 ***	−0.23 ***	−0.20 **	−0.07
Self-esteem	−0.20 **	−0.18 **	−0.12	0.09	−0.25 ***	−0.27 ***	−0.18 **	0.07
Masculine/instrumental trait	0.03	−0.04	0.13	−0.04	0.02	−0.04	0.07	−0.01
Feminine/expressive trait	−0.19 **	−0.18 **	−0.14 *	−0.07	−0.33 ***	−0.34 ***	−0.23 ***	−0.14 *
Traditional gender role attitudes	0.19 **	0.20 **	0.11	0.02	0.25 **	0.27 ***	−0.18 **	−0.07
**Men with former partner**								
Age	0.16 *	0.18 *	0.06	0.04	0.05	0.07	0.01	−0.03
Educational level ^a^	0.03	0.00	0.02	−0.04	−0.03	−0.01	−0.10	−0.06
Somatic symptoms	0.08	0.12	−0.02	0.03	0.04	0.15 *	−0.11	−0.08
Anxiety and insomnia symptoms	0.00	0.03	−0.03	−0.05	0.03	0.12	−0.08	−0.07
Social dysfunction	−0.10	−0.11	−0.08	−0.01	−0.08	−0.02	−0.11	−0.04
Severe depression symptoms	0.00	−0.02	0.04	0.01	−0.02	−0.02	−0.01	−0.03
Life satisfaction	−0.11	−0.10	−0.09	−0.07	−0.07	−0.08	−0.03	0.04
Self-esteem	−0.09	−0.06	−0.07	−0.15 *	−0.19 *	−0.14	−0.14	−0.09
Masculine/instrumental trait	0.16 *	0.15 *	0.11	0.10	0.10	0.06	0.08	0.12
Feminine/expressive trait	−0.13	−0.09	−0.17 *	0.00	−0.16 *	−0.08	−0.17 *	−0.06
Traditional gender role attitudes	0.21 **	0.20 **	0.16 *	0.02	0.30 ***	0.27 ***	0.15 *	0.20 **

Notes: ^a^ = correlation coefficient calculated with Spearman’s *Rho*; statistically significant correlation coefficients are shown in bold. * *p* < 0.05; ** *p* < 0.01; *** *p* < 0.001.

**Table 9:** Summary of the hierarchical regression results for violence victimization for women’s and men’s groups.

	Women with Current Partner			Women with Former Partner			Men with Current Partner			Men with Former Partner		
	Model 1	Model 2	Model 3	Model 1	Model 2	Model 3	Model 1	Model 2	Model 3	Model 1	Model 2	Model 3
	β	β	β	β	β	β	β	β	β	β	β	β
Age	−0.03	−0.01	−0.04	−0.05	−0.05	−0.01	−0.02	−0.01	0.01	0.18 *	0.15 *	0.14 *
Masculine/ instrumental trait		0.05	0.02		−0.18 *	−0.20 **		−0.04	−0.07		0.22 **	0.10
Feminine/ex-pressive trait		−0.13 *	0.02		0.06	0.11		−0.07	0.09		−0.14	−0.06
Traditional gender role attitudes		0.20 ***	0.09 *		−0.05	0.07		0.18 *	0.08		0.22 **	0.05
Violence perpetration			0.67 ***			0.54 ***			0.58 ***			0.57 ***
*R* ^2^	0.001	0.059	0.479	0.002	0.033	0.317	0.000	0.048	0.338	0.033	0.125	0.408
*R*^2^ Change	0.001	0.059 ***	0.419 ***	0.002	0.031	0.284 ***	0.000	0.047 *	0.290 ***	0.033 *	0.091 **	0.283 ***

Notes: β = standardized regression coefficient; *R*^2^ = percentage of explained variance; statistically significant β coefficients are shown in bold; * *p* < 0.05; ** *p* < 0.01; *** *p* < 0.001.

**Table 10:** Summary of the hierarchical regression results for violence perpetration for women’s and men’s groups.

	Women with Current Partner			Women with Former Partner			Men with Current Partner			Men with Former Partner		
	Model 1	Model 2	Model 3	Model 1	Model 2	Model 3	Model 1	Model 2	Model 3	Model 1	Model 2	Model 3
	β	β	β	β	β	β	β	β	β	β	β	β
Age	0.06	0.08	0.11	−0.08	−0.07	−0.06	−0.02	−0.02	−0.01	0.07	0.03	−0.03
Masculine/ instrumental trait		0.09	0.06		0.01	0.10		0.01	−0.02		0.18 *	0.05
Feminine/ex-pressive trait		−0.20 **	−0.13 **		−0.13	−0.18 **		−0.24 **	−0.14 **		−0.14	−0.06
Traditional gender role attitudes		0.17 ***	0.04		0.03	0.06		0.19 **	0.10 *		0.30 ***	0.20 **
Violence victimization			0.61 ***			0.49 ***			0.66 ***			0.54 ***
*R^2^*	0.003	0.072	0.424	0.006	0.023	0.253	0.000	0.126	0.537	0.005	0.134	0.392
*R^2^* Change	0.003	0.069 ***	0.352 ***	0.002	0.017	0.231 ***	0.000	0.125 ***	0.412 ***	0.005	0.129 ***	0.258 ***

Notes: β = standardized regression coefficient; *R*^2^ = percentage of explained variance; statistically significant β coefficients are shown in bold; * *p* < 0.05; ** *p* < 0.01; *** *p* < 0.001.

## Data Availability

Data are contained within the article.
